# The results of a unique dietary supplement (nutraceutical formulation) used to treat the symptoms of long-haul COVID

**DOI:** 10.3389/fnut.2022.1034169

**Published:** 2022-10-25

**Authors:** Norman B. Gaylis, Ida Kreychman, Joanne Sagliani, Josef Mograbi, Yankel Gabet

**Affiliations:** ^1^The COVID Long Haul Center at Arthritis & Rheumatic Disease Specialties (AARDS), Miami, FL, United States; ^2^Department of Anatomy and Anthropology, Sackler Faculty of Medicine, Tel Aviv University, Tel Aviv-Yafo, Israel; ^3^Independent Researcher, Tel Aviv-Yafo, Israel

**Keywords:** dietary (food) supplements, long-COVID-19, immunology and inflammation, cannabinoids, CB2 agonists

## Abstract

Long-COVID is a syndrome characterized by debilitating symptoms that persist over 3 months after infection with the SARS-CoV-2 virus. It affects 15 to 33% of COVID-19 recovered patients and has no dedicated treatment. First, we found that β-caryophyllene and pregnenolone have a significant synergistic effect in the resolution of LPS-induced sepsis and inflammation in mice. Then we combined these two compounds with seven others and designed a unique dietary supplement formulation to alleviate long COVID inflammatory and neurological disorders. We performed a one-arm open-labeled study at a single site with 51 eligible patients from 18 states. Each participant recorded the severity level of 12 symptoms (including fatigue, weakness, cardiac and neurological symptoms, shortness of breath, gastrointestinal disorders, ageusia or anosmia, anxiety, joint pain, rash, cough, and insomnia) at baseline, 2- and 4-week time points. On average, all the symptoms were significantly milder after 2 weeks, with further improvement after 4 weeks. Importantly, each symptom was significantly attenuated in 72 to 84% of the participants. There were no significant adverse effects. Our data indicate that the use of this nutraceutical product is a safe and significantly efficient option to reduce multiple symptoms of long COVID.

## Introduction

Long-haul COVID is characterized by chronic and often debilitating symptoms following acute Coronavirus disease 2019 (COVID-19) caused by the SARS-CoV-2 virus. Long COVID is very challenging to diagnose, treat and categorize as it combines multiple and different symptomatic presentations in sufferers.

The Center for Disease Control (CDC) in June 2022 published a report that 40% of adults in the US reported having COVID-19 in the past, of which nearly 1 in 5 are still having symptoms of long COVID. The CDC defines Long COVID as symptoms that last three or more months after first contracting the virus and did not exist before the COVID-19 infection. Symptoms may last well over 1 year (1). Other studies reported a prevalence ranging from 1 in 8 to 1 in 3 (2, 3), significantly higher than after influenza (2). There appeared to be differences in the prevalence of long COVID between states (2).

Differences in the reported prevalence of long-COVID likely results from the disparity of symptoms and the lack of a unified definition for this condition. Importantly, this condition cannot be ignored as it presents a set of symptoms that often severely impact the quality of life and ability to carry out daily activities. For example, in one survey, 44% percent of patients with Long COVID reported not being able to work at all, compared to their pre-COVID-19 work capacity, and 51% had reduced their working hours (4). Overall, the economic burden may approximate $50 billion annually in lost salary only (4).

An international cohort of 3,762 participants from 56 countries identified 203 symptoms in 10 organ systems that persisted at least 4 weeks after a confirmed diagnosis of COVID-19 (5). The CDC listed the most common symptoms of COVID-19 in a survey they initiated in April 2022.^1^ These included tiredness or fatigue, difficulty thinking, concentrating, forgetfulness, or memory problems (sometimes referred to as “brain fog”), difficulty breathing or shortness of breath, joint or muscle pain, fast-beating or pounding heart (also known as heart palpitations), chest pain, dizziness on standing, menstrual changes, changes to taste/smell, or inability to exercise.

The exact underlying cause of long-COVID remains uncertain, but most reports agree that this condition is associated with a persistent viral infection and long-lasting inflammation (6, 7). Specific neurological symptoms (fatigue, brain fog, anosmia, and ageusia/dysgeusia) in long COVID resemble “sickness behavior,” a response of the autonomic nervous system to pro-inflammatory cytokines (8). The long-standing dysautonomia has been proposed to result from sympathetic/parasympathetic imbalance (7, 9).

To the best of our knowledge, no medicine is currently dedicated to treating long-haulers (patients suffering from long-COVID). In the absence of established protocols, each symptom is treated separately with different drugs for different symptoms, even though they all have a common cause. Even when combining the current therapeutic approaches with rehabilitation (10–14), success is minimal, and the treatments are associated with multiple adverse effects.

We thus designed a formula to address the underlying chronic inflammatory status and autonomic imbalance that characterize many long-haulers. The first challenge was to help restore the function of various organ systems; the second challenge was to use only US-recognized dietary supplements to be able to address the unmet need in a very short time.

Tel Aviv University, in collaboration with Arthritis & Rheumatic Disease Specialties (AARDS) based in Miami, FL, USA, formulated a unique oral nutraceutical supplement containing only approved dietary supplements and ingredients “generally recognized as safe” (GRAS) by the United States Food and Drug Administration (FDA). This combination would, by design, be natural, free of any known side effects, and with limited drug interactions. The final formulation relied on new experimental data (presented here), clinical experience in treating long-hauler patients at AARDS with multiple clinical symptoms over 2 years, and scientific literature (Table 1). We selected the ingredients for their unique immuno-modulation properties and activities, reduction in pain, anxiety and depression, potential effect on dysautonomia, anticoagulation activity, as well as the ability to inhibit viral replication. We report here the results of a clinical study aimed at testing this nutraceutical formula as a standalone treatment in a population of COVID long haulers.

**TABLE 1:** List of compounds included in our nutraceutical combination and their relevance for COVID long haulers.

Active ingredient	Properties and activities	References
β-caryophyllene (βCP)	Antioxidant, anti-inflammatory, analgesic	(18, 27–29)
Pregnenolone	Modulator of inflammation, regulator of neuroinflammation	(22, 30–32)
Dehydroepiandrosterone (DHEA)	Anti-inflammatory, improves dysfunction of the hypothalamic-pituitary-adrenal (HPA) axis, immune modulator	(33–35)
Bromelain	Anti-inflammatory, antiviral (Anti-SARS-CoV-2), fibrinolytic	(36–39)
St. John’s Wort extract	SRI* for treating autonomic dysfunction and impaired balance between the sympathetic and parasympathetic nervous systems	(40, 41)
Boswellia Serrata gum/resin extract (AKBA)	Anti-inflammatory, COVID-19 therapeutic agent, respiratory support	(42–44)
Quercetin	Anti-inflammatory, antiviral, immune modulator, antioxidant	(45–47)
Zinc compound	Essential to preserve natural tissue barriers such as the respiratory epithelium, balanced function of the immune system and the redox system, antiviral	(48, 49)
Vitamin D	Reducing inflammation induced by SARS-CoV-2 infection	(50, 51)

*SRI, serotonin reuptake inhibitor.

We and others have reported that selective CB2 agonists are potent immunomodulators of the innate immune system, both *in vivo* and *in vitro* (15, 16). We showed that CB2 agonists inhibit cytokine expression in LPS-exposed macrophages in cultures and decrease ear edema in a skin inflammation model in mice (16). Following these results, we tested the hypothesis that β-caryophyllene (βCP), a dietary terpene that is also a selective CB2 agonist (17, 18), reduces the exaggerated inflammatory response induced by pathogens.

Another approach to attenuate inflammation and associated tissue damage is using steroids. In contrast to glucocorticosteroids such as dexamethasone, pregnenolone is a steroid hormone precursor with known anti-inflammatory properties in myeloid cells (19), does not induce lymphocyte apoptosis (20) and may even promote thymocyte survival and differentiation (21). Pregnenolone (Preg) and its metabolites suppress the secretion of tumor necrosis factor α and interleukin-6 mediated through TLR2 and TLR4 signaling in macrophages (22). Also relevant to long-haulers, Preg can help with cognitive and neurological issues, partly *via* binding to TRPM3, without causing bone loss (23–26).

In addition to βCP and Preg, we included Dehydroepiandrosterone (DHEA), Bromelain, St. John’s Wort extract, Boswellia Serrata gum/resin extract (AKBA), Quercetin, zinc compound, and vitamin D. The rationale for including each compound is summarized in Table 1.

## Materials and methods

### Mouse study

Male wild type 8–10 weeks old mice of inbred strain C57BL/6J-RCC were obtained from Envigo Ltd (Jerusalem, Israel). All experiments were in accordance and with the approval of the institutional animal care and use committee of Tel-Aviv University for these experiments (permit number 01-20-022). The mice were divided into treatment groups 24 h before LPS administration. All treatments were administered intraperitoneally (IP). β-Caryophyllene (βCP) and pregnenolone were injected every 12 h starting 24 h before the LPS injection. A single IP injection of LPS was administered at 25 μg/gr dose. This study had two control groups as indicated in the experiments below. One that received PBS instead of the LPS injection (“PBS”), and one that received PBS instead of the treatments (CB2 agonist or steroid) before the LPS injection (“LPS”). For humane reasons murine sepsis score (MSS) was used as a surrogate for survival (52). A low MSS score (0–2) served as a surrogate for survival while mice that reached the score 3 were defined as critically ill and were euthanized.

### Statistical analyses for the animal study

For survival experiments in mice, Gehan–Breslow–Wilcoxon test was used for multiple groups comparison. For disease progression we used Dunnett’s multiple comparisons test. Differences between groups were considered significant when *p* < 0.05.

### Clinical study performed at arthritis and rheumatic disease specialties

The new combination of nutraceuticals including the compounds listed in Table 1 was tested in a one-arm, open-label clinical trial. The amount of each compound in the formulation and the total amount per serving are detailed in Table 2. Participation was voluntary, recruitment was from local patient population as well as social media advertising. All subjects had to have documented evidence of prior COVID infection (PCR or Rapid test) and were having ongoing symptoms (1 or more) for a minimum of 3 months from the time of infection. The existence of any of these symptoms prior to contracting COVID was considered an exclusion. All subjects were required to complete a survey listing and rating their symptoms and all participants were interviewed by the research staff at baseline to verify inclusion criteria.

**TABLE 2:** Amount of each compound included in the nutraceutical formulation per serving. The participants were required to take one serving twice a day with food.

Active ingredient	Amount per serving
β-caryophyllene (βCP)	40 mg
Pregnenolone	40 mg
Dehydroepiandrosterone (DHEA)	30 mg
Bromelain (2400 GDU*/g)	416 mg
St. John’s Wort extract	150 mg
*Boswellia Serrata* gum/resin extract (AKBA)	100 mg
Quercetin (*Sophora Japonica*)	40 mg
Zinc (as Zinc Picolinate)	12 mg
Vitamin D	25 μg (1000 IU)

*GDU, gelatin digesting unit.

Following qualification, subjects were provided with the nutraceutical supplement for a 2-week period with dosing instructions (1 serving twice a day with food) after which they were required to repeat the process of documenting any change in symptoms relative to baseline and their voluntary decision to continue with treatment for a further 2-week period. At the end of 4 weeks, their change in symptoms from baseline was documented as well as an overall global response.

The total number of patients completing the 4-week evaluation was 51, the age range was 21–73 years of age, female to male ratio was approximately 2 to 1 and the patients originated from 18 US states.

### Statistical analysis of the clinical study

We compared the score of each symptom at 2 and 4 weeks to the baseline for each participant using multiple paired *t*-tests. Individual variance was assumed for each symptom and a Benjamini–Krieger–Yekutiel False Rate Discovery Rate (FDR) was used to account for the multiple comparisons. For all comparisons, differences between time points were considered significant when *p* < 0.01.

## Results

### Animal study

#### Testing of anti-inflammatory compounds in mice

**FIGURE 1:**
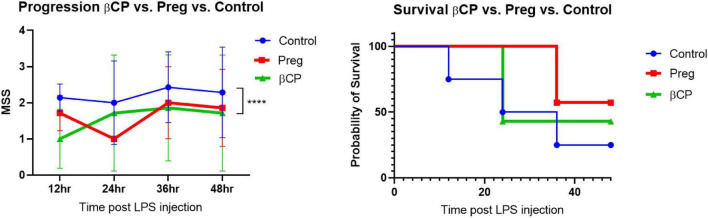
All groups were injected with LPS 25 μg/g. Mice were pre-treated with β-caryophyllene (BCP, 25 mg/kg) or Pregnenolone (Preg, 10 mg/kg) 24 and 12 h before LPS injection and then every 12 h. *N* = 7. ****p < 0.0001, 2-way ANOVA between βCP and Controls.

**FIGURE 2:**
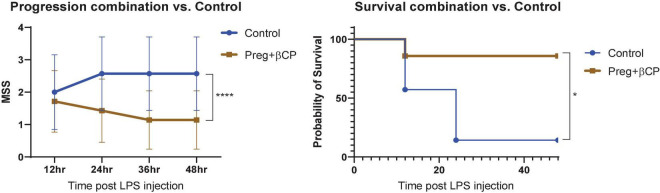
All groups were injected with LPS 25 μg/g. Mice were pre-treated with β-caryophyllene (BCP, 25 mg/kg) and Pregnenolone (Preg, 10 mg/kg) 24 and 12 h before LPS injection and then every 12 h. *N* = 7. ****p < 0.0001, 2-way ANOVA for disease progression; *p = 0.0152, Log-rank (Mantel-Cox) test for survival.

Here we tested the therapeutic potential of β-caryophyllene and that of pregnenolone in a mouse model of LPS-induced sepsis. βCP (25 mg/kg) and Preg (10 mg/kg) were administered 24 and 12 h before LPS injection (25 mg/kg), and then every 12 h. All the injections were given i.p. A Murine Septic Score (MSS) was recorded every 12 h and survival was defined as MSS < 3. Over the 4-day follow-up, we found a slight beneficial effect of βCP in improving disease progression (improved “well-being”), while Preg alone had no significant effect (Figure 1). When measuring survival, both compounds induced a slight decrease in mortality, but none had a statistically significant effect (Figure 1). Next, we asked whether the combined treatment with both compounds can improve the outcome of the mice to LPS injection. Our results show that combining βCP and Preg had a significant positive effect on both disease progression and survival (*p* = 0.026, Figure 2). Indeed, the combined treatment with βCP and pregnenolone significantly improved the wellbeing by >twofold and the survival from a non-significant effect to a >90% increase (Figure 2). These data demonstrate that βCP and Preg have a synergistic effect in alleviating inflammation as well as disease severity and mortality from sepsis *in vivo*.

Following the demonstration of a synergistic effect of βCP and Preg in the resolution of inflammation, we combined these two compounds with seven additional supplements. We designed the resulting formulation to address the symptoms of long-COVID, related to persistent viral infection, long-lasting inflammation and neurological disorders.

### Clinical trial

#### Clinical study on COVID long hauler patients

**FIGURE 3:**
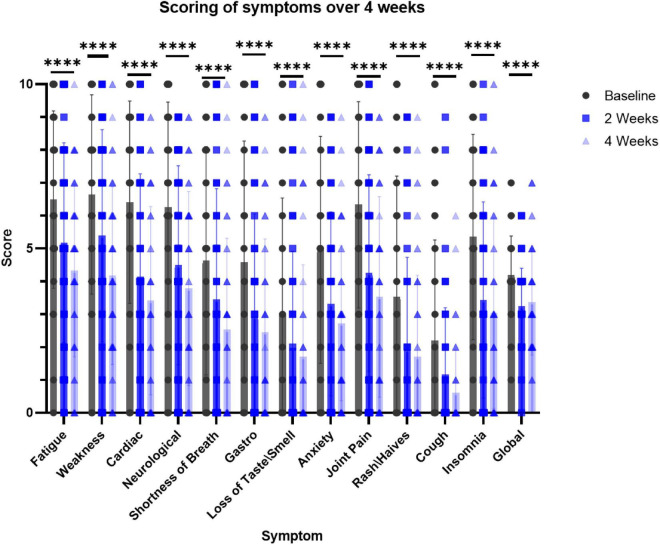
Survey-based scoring by participants at the intake to the trial (baseline), 2 and 4 weeks while taking the supplement. *N* = 51. Dunnett’s multiple comparisons test, *p* < 0.0001 in 2 and 4 weeks for the effect of the supplement on all the symptoms (vs. baseline). ^****^p < 0.006 paired *t*-test, at 2 weeks vs. baseline and 4 weeks vs. baseline for each symptom.

At baseline, 2- and 4-week time points, participants recorded the level of severity for each of the 12 symptoms in addition to their subjective assessment of wellbeing (”global reported”). The symptoms in the survey included fatigue, physical weakness, cardiac (e.g., palpitations) and neurological symptoms (e.g., “brain fog”), shortness of breath, gastrointestinal disorders, loss of smell or taste, anxiety, joint pain, rash or hives, cough and insomnia. All the participants took the recommended daily dose of the nutraceutical for 4 weeks. The surveys and scoring by the participants revealed that the severity score for all the symptoms improved already after 2 weeks and this beneficial effect tended to be even more pronounced after 4 weeks (Figure 3). Notably, the decrease in the severity level of all the symptoms was statistically significant at both the 2- and 4-week time points.

**FIGURE 4:**
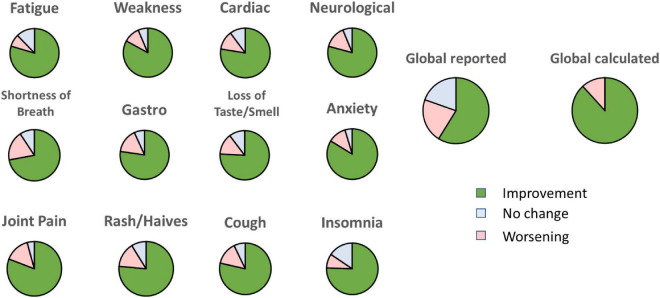
Percentage of participants reporting an improvement, no change or worsening for each symptom at week 4 relative to baseline. “Global reported” is the score given by each participant on general feeling. “Global calculated” is the average of all the scores for each symptom.

Next, we evaluated the number of symptoms that improved for each participant. The vast majority of the participants (46 out of 51) reported more symptoms that improved than symptoms that worsened. Among the remaining five participants, only one reported feeling a worsening in general wellbeing. We also calculated the percentage of participants who reported an improvement, worsening, or no change for each symptom over the 4-week treatment. For each symptom, we omitted any participants who reported a null severity score (”zero”) for this symptom at all time points as these do not denote a lack of effect. Notably, 72 to 84% of the participants reported an improvement for each of the 12 symptoms (Figure 4). When asked about their general wellbeing (”global reported”), 59% reported a noticeable improvement. However, when we calculated the average score of all the symptoms for each participant (”global calculated”), we found that the general profile improved for 88% of the participants.

#### Safety data

Overall safety data was very good, and no major adverse events were noted. Minor adverse events included three patients with vertigo, one patient with increased anxiety, one patient with a gout attack, one patient with increased joint pain and one patient was reinfected with COVID.

## Discussion

The results suggest this unique nutraceutical dietary supplement combination may afford significant symptomatic benefit to long COVID sufferers. The adverse events were minor and overall safety of the nutraceutical product was confirmed.

As the clinical study results indicate, there were statistically significant improvements in this study population in their overall symptoms after 2 and 4 weeks of treatment. It should be noted that different symptoms improved or worsened in different patients. There were some symptoms such as fatigue and brain fog that appeared to respond more than others, however, no baseline presentations were able to predict individual symptomatic responses.

It is unknown at this time if the duration of benefit will be complete, short term, or long term. Because the follow-up period ended after 4 weeks and treatment was taken until the last day, this study was specifically designed to demonstrate a beneficial effect on the long-COVID symptoms. Further studies are warranted to determine the optimal duration of the treatment and whether symptoms would recur upon cessation of the treatment.

We observed a discrepancy between the sum of improvements for each symptom and the reported global wellbeing. Indeed, calculating the average improvement of all the symptoms for each individual, 88% of the participants benefited from the treatment; however, only 59% of the participants reported an overall improvement of their wellbeing (Figure 4). A similar discrepancy has been reported by others in post-stroke patients where general wellbeing was poorly associated with changes in executive function and comorbidities (53). In our study, we may speculate that not all participants perceived all symptoms at the same level. For example, a person experiencing an improvement in 4 out of 5 symptoms may still consider no improvement in overall wellbeing if the one unchanged symptom has a severe impact on his or her quality of life.

Our *in vivo* experiment describes for the first time the synergistic effect of two different compounds in the attenuation of systemic inflammation. We could find no report on a putative interaction between βCP and Preg. However, a study showing that Preg may act as an allosteric modulator of CB1 (54), a receptor that shares a 44% homology with CB2 (55), may provide circumstantial evidence to a similar interaction between CB2 activation and Preg. In addition to βCP and Preg that have potent anti-inflammatory role, we selected nutraceutical compounds that have been tested and proven effective as anti-viral and/or immunomodulatory agents (DHEA, Bromelain, AKBA, Quercetin, Zinc, and Vitamin D) or in managing neurological dysfunctions (DHEA, St John Wort, and Zinc, see Table 1). Further studies may be warranted to elucidate the relative contribution of each of the nine compounds included in this nutraceutical to the various symptoms of Long-COVID.

This product is formulated for adults excluding pregnant and breastfeeding women. It is also contra-indicated in combination with serotonin-related antidepressants medications due to the St. John’s Wort extract. When indicated, this new nutraceutical is a safe product to be used in combination with other standard of care therapies prescribed for long COVID. We emphasize that the product is not meant to replace standard recommendations of treatment, vaccinations or other suggested methods of prevention or treatment of COVID-19.

In this study, we compared the treatment outcome after 2 and 4 weeks to the same participant’s baseline. While there was no placebo in this clinical study, every patient had tried and failed numerous nutraceutical and pharmaceutical products that were available to them along 3–20 months before the trial with no success. In addition, eligibility included persistence of the symptoms for at least 3 months, which reduces the likelihood that the positive effects are coincidental.

The use of nutraceuticals for specific long-COVID symptoms has been previously suggested (56). Here, we developed a nutraceutical for the treatment of a large array of long-COVID symptoms and we demonstrated statistically significant improvement in all tested endpoints in a relatively short time. Within the limitations of this study, our data indicate that the use of this nutraceutical product is a safe and significantly efficient option to reduce multiple symptoms of long COVID. To the best of our knowledge, our population study represents the largest group of patients to have shown statistically significant symptomatic improvement to a nutraceutical formulation.

## Patents

Patent pending, International Patent Application No. PCT/IL2022/050676.

## Data availability statement

The original contributions presented in the study are included in the article/supplementary material, further inquiries can be directed to the corresponding authors.

## Ethics statement

Ethical review and approval was not required for the study on human participants in accordance with the local legislation and institutional requirements. The patients/participants provided their written informed consent to participate in this study. The animal study was reviewed and approved by the Tel Aviv University Animal Studies Ethics Committee.

## Author contributions

NG, JM, and YG: conceptualization, writing – original draft preparation, and project administration. NG and YG: methodology, resources, supervision, and funding acquisition. NG, IK, JS, and YG: validation and investigation. IK, JS, and YG: formal analysis. JS and IK: data curation. IK and YG: writing – review and editing and visualization. All authors reviewed and approved the final version.

## Footnotes

1
cdc.gov
